# Could biochar amendment be a tool to improve soil availability and plant uptake of phosphorus? A meta-analysis of published experiments

**DOI:** 10.1007/s11356-021-14119-7

**Published:** 2021-05-08

**Authors:** Fitsum Tesfaye, Xiaoyu Liu, Jufeng Zheng, Kun Cheng, Rongjun Bian, Xuhui Zhang, Lianqing Li, Marios Drosos, Stephen Joseph, Genxing Pan

**Affiliations:** 1 Institute of Resource, Ecosystem and Environment of Agriculture, Nanjing Agricultural University, 1 Weigang, Nanjing, 210095 Jiangsu China; 2 Department of Soil Science, Nanjing Agricultural University, 1 Weigang, Nanjing, 210095 Jiangsu China; 3 Jiangsu Collaborative Innovation Center for Solid Organic Waste Resource Utilization, Nanjing Agricultural University, 1 Weigang, Nanjing, 210095 Jiangsu China

**Keywords:** Biochar, Soil amendment, Phosphorus, Soil availability, Plant uptake, Agricultural soil, Meta-analysis

## Abstract

As one of the most important nutrients for plant growth, phosphorus was often poorly available in soil. While biochar addition induced improvement of soil structure, nutrient and water retention as well as microbial activity had been well known, and the effect of biochar soil amendment (BSA) on soil phosphorus availability and plant P uptake had been not yet quantitatively assessed. In a review study, data were retrieved from 354 peer-reviewed research articles on soil available P content and P uptake under BSA published by February 2019. Then a database was established of 516 data pairs from 86 studies with and without BSA in agricultural soils. Subsequently, the effect size of biochar application was quantified relative to no application and assessed in terms of biochar conditions, soil conditions, as well as experiment conditions. In grand mean, there was a significant and great effect of BSA on soil available P and plant P uptake by 65% and 55%, respectively. The effects were generally significant under manure biochar, biochar pyrolyzed under 300 °C, soil pH <5 and fine-textured soil, and soils that are very low in available P. Being significantly correlated to soil P availability (*R*^*2*^=0.29), plant P uptake was mostly enhanced with vegetable crops of high biomass yield. Overall, biochar amendment at a dosage up to 10 t ha^−1^ could be a tool to enhance soil availability and plant uptake of phosphorus, particularly in acid, heavy textured P-poor soils.

**Supplementary Information:**

The online version contains supplementary material available at 10.1007/s11356-021-14119-7.

## Introduction

Soil phosphorus (P) had been a major limiting factor for crop production on many tropical and subtropical soils (Norman et al. 1995) and a continuous utilization at large amount of soil P by crops would eventually lead to a rapid depletion of available phosphorus pool in soil (Vuuren et al. 2010). The amount of readily available phosphorus was generally low, ranging from 0.1 to 10 μM (Hinsinger 2001), compared to the relatively large stock of total phosphorus in soil (Adnan et al. 2003; Batjes 2011). Furthermore, crop uptake and fraction of the fertilizer P that was directly taken up by plant roots governed the level of available P present in soil (Mogollón et al. 2018). Phosphorus supply in agricultural soils impacted global food systems by ensuring soil fertility, farmer livelihoods, agricultural productivity, and global food security (Cordell and White 2015).

Availability of soil P was basically very closely correlated to soil pH (Adnan et al. 2003) but soil P supply to crops depends also on P-status of the parent material and its management along the farming history (Batjes 2011). Phosphorus could be available to plants mostly in a soil pH range of 6 to 7 (Ch'ng et al. 2014) as phosphorus tended bound with Al and Fe at low pH but with Ca and Mg at high pH (Chintala et al. 2014) in immobilized forms unavailable to plant. Phosphorus immobilization was strong in acidic soils where phosphorus deficiency mostly observed (Adnan et al. 2003). Globally, croplands with P deficiencies were estimated at 5.7 billion ha (67% of total croplands), with severe phosphorus constraints for sustaining crop production particularly in subtropical and tropical regions with highly weathered soils of the world (Hinsinger 2001; Zhang 2016). Consequently, P fertilizers were applied at approximately 15 million tons each year to maintain the P supply to crops in world agriculture (Wang et al. 2012).

Phosphorus derived mainly from mined rock phosphate as a non-renewable resource. P fertilizer demand would increase as the world population with changing diets was projected to increase by 50% over the next 50 years (Cordell 2008). Owing to the decreasing stock, the price of rock phosphate increased by folds in recent decades (Cordell and Neset 2014). For future global food security under a great challenge of the phosphorus resource scarcity, phosphorus should be recovered for productive reuse as a fertilizer in food production to replace increasingly scarce and expensive phosphate rock (Cordell et al. 2011). Thus, a “quick fix” solution to tackle mineral phosphorus scarcity had been urged (Cordell 2008). As such, reuse of low cost and readily available animal wastes and municipal bio-solids as a potential P source had been generally encouraged to recover nutrients, manage waste, and improve soil fertility (Cui et al. 2011;Kleinman et al. 2001; Wang et al. 2012). Conversion of waste biomass to biochar through pyrolysis had more advantages than the direct application to soil (Wang et al. 2012).

Biochar was considered a carbonaceous material obtained by biomass pyrolysis (Conte 2014), at temperatures generally lower than 700°C in limited presence of oxygen (Lehmann and Joseph 2009). Varying with feedstocks and pyrolysis conditions (Uchimiya et al. 2015; Lehmann et al. 2015; Dai et al. 2016), physical and chemical properties of biochar were the keys to understanding the performances and mechanisms of biochar in the improvement of soil fertility (Jeffery et al. 2011). Application of biochar to soil had been shown significant effects on increasing nutrient availability (Sohi et al. 2010; Xu et al. 2013; Glaser and Lehr 2019), enhancing plant growth (Zhang et al. 2012; Ahmed and Schoenau 2015; Blackwell et al. 2015; Kelly et al. 2015; Brantley et al. 2016; Li et al. 2016; Amin and Eissa 2017; Si et al. 2018) except for elevating soil pH (Alburquerque et al. 2013; Ding et al. 2016).

The effects of biochar on P availability in soil were related to P level and capacity of P sorption and desorption either of soil (Farrell et al. 2014; Xu et al. 2014; Zhang et al. 2016a) or of biochar (Borno et al. 2018). Further, the increased nutrient availability to plants with biochar could be due to the direct nutrient addition from amended biochar and to indirect changes in nutrient retention against leaching (Madiba et al. 2016) and in microbial P turnover (Lehmann et al. 2006) in amended soil. For example, increases in P availability with increasing biochar application rates were often observed with pH rise in acidic soils (Chan et al. 2007; Atkinson et al. 2010; Blackwell et al. 2010; Laird et al. 2010; Peng et al. 2011; Jones et al. 2012; Biederman and Harpole 2013; Yuan and Xu 2012) in addition to direct P supply with biochar (Chan et al. 2007; Atkinson et al. 2010; Laird et al. 2010; Ch'ng et al. 2014). Furthermore, plant P uptake could also be changed with biochar application owing to direct change in available P level and to indirect change in soil environment for microorganisms (Atkinson et al. 2010; Laird et al. 2010) and for root growth (Lehmann et al. 2011). Such changes were responsible for increasing nutrient mobilization and uptake in the rhizosphere via improving the exploratory capacity of root system and modifying nutrient solubility (Alburquerque et al. 2013; Lehmann et al. 2011). Yet, there had not been a general understanding how biochar affects soil available P level and plant P uptake in agricultural soils though biochar had been early characterized and evaluated for use in agriculture (Singh et al., 2010).

Therefore, it is hypothesized that soil available P level and plant P uptake could be improved with biochar application either due to P enhancement in soil or due to soil condition changes. The former would be biochar type and dose dependent while the later soil type dependent. In this study, we performed a comprehensive meta-analysis of published experiments that tested the effects of biochar amendment on soil available phosphorus and the uptake by plants. Factors related to changes in soil P availability following biochar amendment were explored in terms of biochar properties (feedstock type, pyrolysis temperature, and application dose), experiment type and duration, soil properties (soil available P and pH, specifically), and plant type. We aimed to provide strategic insights into biochar’s effect on plant growth and crop yield and into potential biochar technology to improve crop production in global agriculture.

## Material and method

### Literature search

For meta-analysis, literature search was performed using the key words of “biochar,” “biochar soil amendment,” “agricultural soil,” and “phosphorus or P.” Studies, with exclusion of review papers, published by February 1, 2019, were searched via Web of Science, Springer Link, Wiley-Blackwell, and the Chinese magazine network (CNKI) databases. Data were compiled from the literature reporting incubation, field, and pot studies that compared the content of available P in the soil and the total P uptake by the plant in the soil amended with biochar against the biochar un-amended control soil. The originality of data was identified by evaluating the title and abstract of the articles, and those articles that met these criteria were examined in detail. GetData Graph Digitizer 2.26.0.20 software was used to extract numerical data presented in the figures. Finally, 516 data pairs from 86 publications were obtained for analysis. Data regarding the type and duration of the experiment, biochar (feedstock type, pyrolysis temperature, and application rate,), soil (available P, pH, and soil texture), and crop type were also retrieved from the publications.

### Data treatment

Before analysis, data were standardized for comparison. Firstly, biochar dose was all expressed in t ha^−1^. For this, a biochar dose reported in % was converted to t ha^−1^ using the data of bulk density and depth of the studied soil to which biochar was applied. When pyrolysis temperatures were provided as a temperature range, the maximum pyrolysis temperatures were recorded and used. The pH values were recorded as measured in water or converted as pH in water in case measured with CaCl_2_, using the following equation (Gavriloaiei 2012):

1 $$ \mathrm{p}{H}_{water}=0.400+1.028\times \mathrm{p}H\  CaC{l}_2 $$

Soil P availability was recorded as Olsen-P as reported in literature or converted into Olsen-P in case Colwell P, and Bray–1 P or Mehlich–3 P was reported using the following equations suggested by Kleinman et al. (2001):

2 $$ \mathrm{Olsen}\ \mathrm{P}=5.69+0.46\times \mathrm{Mehlich}\hbox{--} 3\ \mathrm{P} $$

3 $$ \mathrm{Olsen}\ \mathrm{P}=11.4+0.44\times \mathrm{Bray}\hbox{--} 1\ \mathrm{P} $$

4 $$ \mathrm{Olsen}\ \mathrm{P}=1.02+0.35\times \mathrm{Colwell}\ \mathrm{P} $$

The original numerical data of soil available P content, soil pH_water_, and plant P (uptake) reported in the studies were recorded directly for calculation of biochar effects. Values of standard deviation of the data reported were directly recorded. In case of standard errors presented, value of standard deviation was calculated by multiplying the standard error by √*n*, where *n* is the number of replicates. In case the variance was not provided, a standard deviation was assigned as 10.12% to the mean, following Luo et al. (2006).

For examining the factors influencing biochar effect, the reported conditions of soil, biochar, and plant were respectively grouped and categorized into classes, basically following Cayuela et al. (2013). In detail, experiment type was grouped as lab incubation, pot, and field experiment, while experiment duration as less than 3 months (≤90 days), 3–6 months (90–180 days), 6–12 months (181–365 days), and more than 12 months (>365 days); biochar feedstock type was grouped as crop residue (straw, grass, corn cob, peanut shell), manure (poultry, swine or cattle), and wood (acacia, pine, mango, willow, municipal wood waste, bark, olive tree pruning) as well as sewage sludge; pyrolysis temperature was grouped as low (≤ 300 °C), medium (300–500 °C), and high (> 500 °C); biochar application dose was grouped as low (< 5 t ha^−1^), medium (5–20 t ha^−1^), high (20-40 t ha^−1^), and very high (≥40 t ha^−1^); soil texture was grouped as coarse (loamy sand, sand, sandy loam), medium (loam, clay loam), and fine (clay, fine loam, silty clay, loamy clay); soil pH before experiment was grouped as very acid (pH <5.0), slightly acid (pH 5.0–6.5), neutral (pH 6.5–8.5), and alkaline (pH>8.5). Soil initial available P level was grouped as very low (<5 mg kg^−1^), low (5–10 mg kg^−1^), medium (10–25 mg kg^−1^), and high (>25 mg kg^-1^), following AgVita Analytical, 2016. Finally, crop type reported in the studies were grouped as cereal crops (rice, wheat, maize, sorghum, barley), pulse crops (soybean, grams, beans cowpea, and lentil), forage crops (berseem, alfalfa, oats, and grass), and vegetable crops (carrot, potato, onion, radish, lettuce, eggplant, tomato, peppers and melons, cabbage, cauliflower, mustard), as per Balasubramanian (2014).

### Meta-analysis

A meta-analysis was conducted to characterize the change in soil P availability and in plant P uptake with biochar soil amendment (BSA, hereafter). Following Borenstein et al. (2009), an effect size was calculated as a natural log-transformed response ratio (RR):

5 $$ \mathrm{RR}=\ln\ \left({\mathrm{X}}_{\mathrm{t}}/{\mathrm{X}}_{\mathrm{c}}\right) $$

where X_t_ and X_c_ represents the mean under BSA and under the control without BSA, respectively. The standard deviation of the mean was used as measures of variance. The effect size by BSA was calculated by a random-effect model, with which the effect size was weighted in inverse proportion to its variance. Experiment groups with fewer than three data pairs were excluded from the analysis. The mean effect size of each group and its 95% confidence interval (CI) were calculated using Excel 2010. However, to test if selected studies were similar enough to warrant combination, the variations in effect sizes were examined before estimating mean effect sizes and their 95% CIs (Hedges et al. 1999).

With meta-analysis, mean percentage changes in soil available P and plant P uptake were used to present the results. A value of relative change (RC) as the percentage change to the mean of the group was obtained by exponentially transforming the response ratio, using an equation as:

6 $$ \mathrm{RC}=\left(\exp\ \left[\mathrm{RR}\hbox{--} 1\right]\right)\times 100 $$

Resultantly, a positive percentage change represents a significant increase in soil P availability or plant P uptake with BSA, or vice versa.

### Data treatment and statistics

Effect size with BSA calculated for a certain group/category was demonstrated in a graph with forest plots. Therein, a line across the horizontal axis represented the range of percentage change of 95% CI for a given experiment group with the mean effect size indicated by the dot in the middle of this line. Also, the numbers of the observed data pairs in each group, on which the Meta-analysis was based, were provided in parentheses. A difference in the RC of soil available P or plant P uptake between two individual groups was considered significant when their CIs did not overlap.

## Results

### Changes in soil P availability and plant uptake: experiment type and duration

**Fig. 1:**
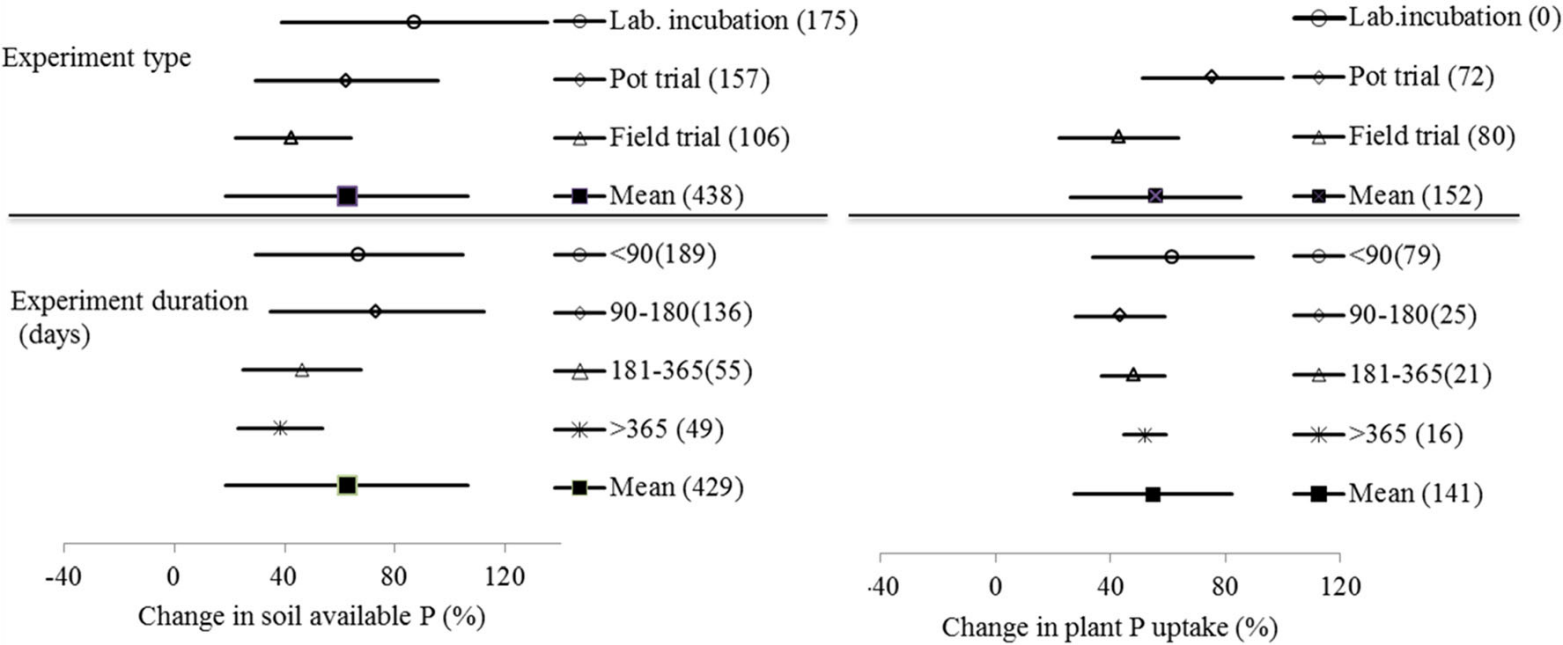
Change in soil available P (left) and plant P uptake (right) under biochar amendment depending on experiment type and experiment duration

Changes in soil available phosphorus (P) with BSA in terms of experiment type and duration are shown in Fig. 1. A grand mean percentage change with BSA was found to be 65% in soil available pool of P and 55% in plant uptake of P respectively, indicating a positive but great BSA effect on soil availability and plant uptake of P in agricultural soils. While variation of the effect sizes was relatively smaller for plant uptake than for soil available pool, there were no significant differences between the experiment groups both on soil available P and plant P uptake. However, percentage changes both in soil available P pool and in plant P uptake were relatively higher in lab or pot experiments than in field and long term experiments.

### Changes in soil P availability and plant uptake: biochar condition

**Fig. 2:**
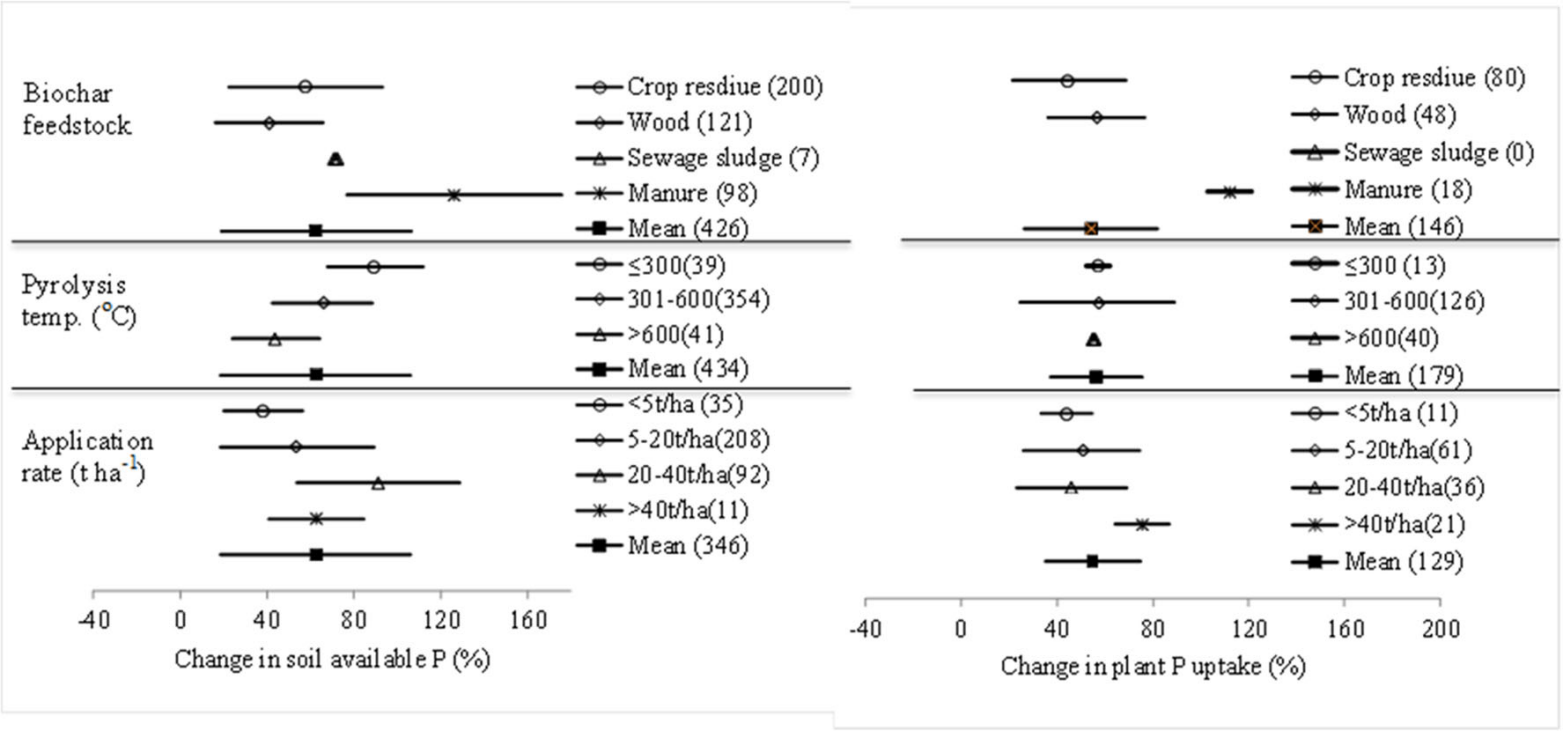
Change in soil available P (left) and plant P uptake (right) under biochar amendment depending on feedstock type of biochar, biochar pyrolysis temperature and application rate

Data of percentage change by BSA in soil available P and in plant P uptake in terms of biochar conditions is presented in Fig. 2. Great variation with biochar conditions of the BSA effect size was found in soil available P rather than in plant P uptake. For biochar feedstock, manure biochar had a greater effect (by over 100%) than biochars of crop residue and wood (by <60%) both on soil available P and plant P uptake. For pyrolysis temperature, whereas, mean percentage change in soil available P was higher with low temperature (≤300 °C) biochars than with high temperature (>600 °C) biochars, while that in plant uptake did not vary much with pyrolysis temperature. However, the effect size on soil available P was more or less proportional to biochar dose but declined when applied in excess (over 40 t ha^−1^). Differently, the effect size on plant uptake was high (by 76% on average) at a dose in a range of 5–40 t ha^−1^, compared to by 44% on average at small dose (< 5 t ha^−1^) and no change at a high dose up to 40 t ha^−1^.

### Changes in soil P availability and plant uptake: soil condition

**Fig. 3:**
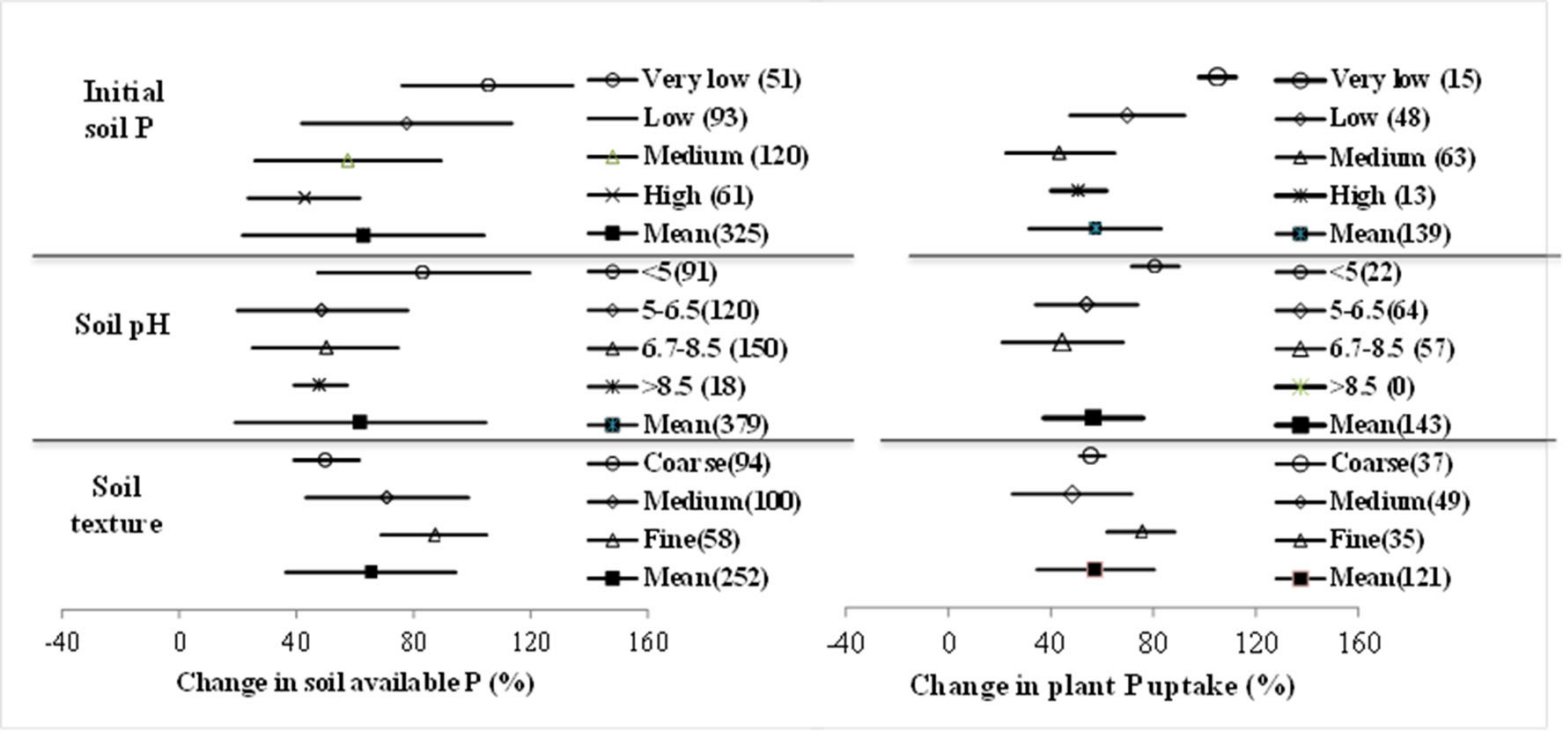
Change in available P (left) and plant uptake (right) under biochar amendment depending on initial soil P, soil pH, and soil texture

In this meta-analysis, the changes in soil available P pool and plant P uptake under BSA in terms of soil condition including initial level of soil P, soil pH, and soil texture are plotted in Fig. 3. For soil available P, the change was increasingly great with decreasing soil available P level and significantly higher in soils poor in available P (by 105% on average) than high in available P (by 42.6% on average). Similarly, the change on average in plant P uptake was by 105% in soils poor in available P, being higher than in soils with medium or high available P (by 44–51%). For soil pH, whereas, the average effect size in soil available P was higher in acid soils (83.5%) than in neutral or alkaline soils (39–50%). Differently, plant P uptake, as increased on average by 81% in acid soils, is being significantly higher than in neutral soils (by 45%). Moreover, the positive change on average in soil available P under BSA was significantly higher in fine textured soils (by 87%) than in coarse-textured soils (by 50%). Similarly, a mean positive change in plant P uptake was of 75% in fine textured soils while of 56% in coarse-textured soils.

### Changes in soil P availability and plant uptake: crop type

**Fig. 4:**
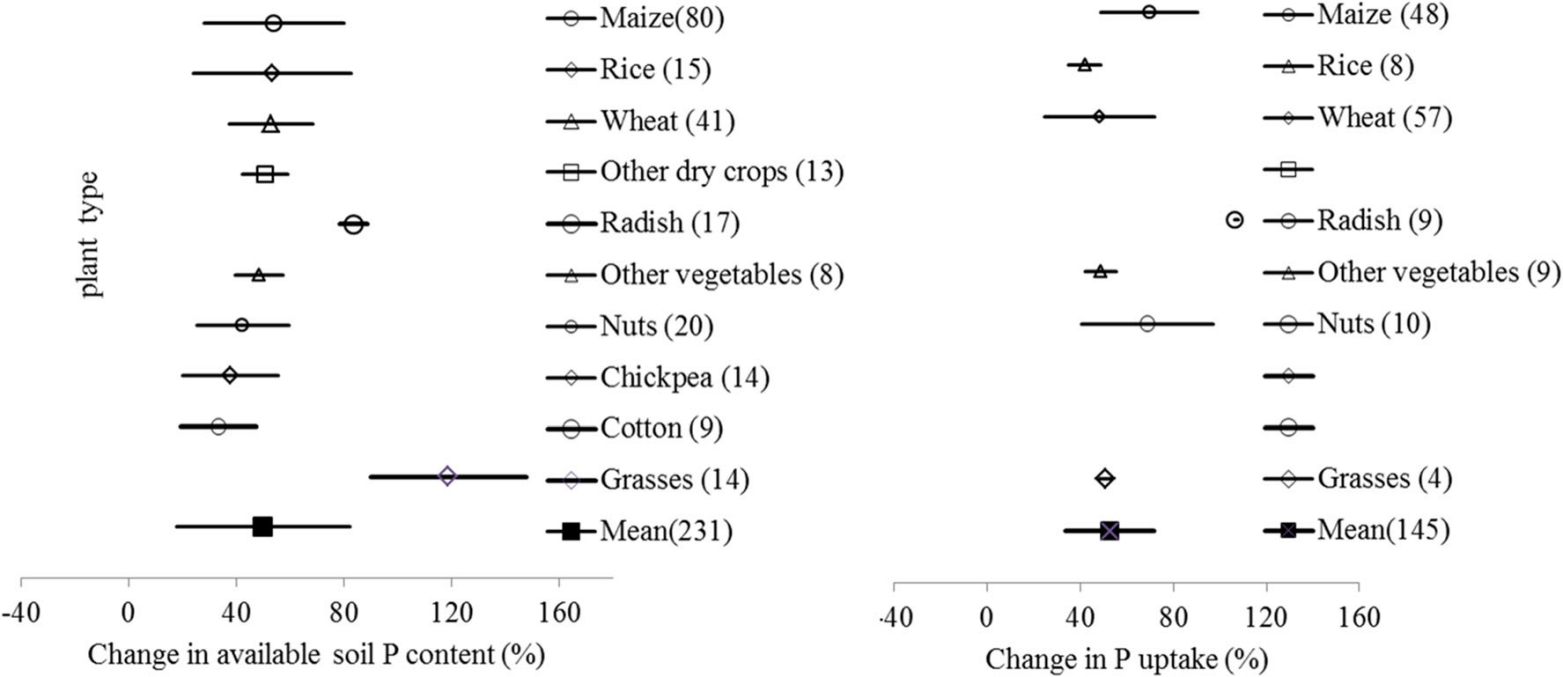
Change in available soil P (left) and plant P uptake (right) under biochar amendment depending on plant/crop types

Data of the percentage changes in soil P availability and plant P uptake related to crop type are exhibited in Fig. 4. For the relative small number of field experiments, data pairs for different crops were much less for plant uptake than for soil available P. Generally, BSA effect sizes on soil available P were similar between crop types, with a grand mean of 50%. The changes tended to be significantly higher under grass for biomass production and radish for root tuber production, despite of fewer cases. With the experiments fewer than soil P studies but mostly for cereals crop of maize and wheat, plant P uptake showed a grand mean of 52.7%, similar to their soil available P change. Yet, radish exerted a high relative change by 108% on average despite 9 cases only.

## Discussions

### Biochar’s role in improving soil available P and plant P uptake

Biochar’s effects had been quantified with meta-analysis on soil physical properties (Omondi et al. 2016), microbial growth (Zhou et al. 2017), and plant growth or crop productivity (Jeffery et al. 2011; Liu et al. 2013). This study revealed a large but consistently positive effect size by BSA both on soil available P pool (by 65% as grand mean) and on plant P uptake (by 55% as grand mean) though the latter in fewer studies. Compared to a low mean effect for crop productivity (by ca 10%, Jeffery et al. 2011; Liu et al. 2013), a medium mean effect for soil microbial growth by 17% (Zhou et al. 2017) and a moderate effect for soil hydrology by 20–25% (Omondi et al. 2016), soil total N by 30% (Biederman and Harpole 2013), metal plant availability reduction by 17–39% (Chen et al. 2018) and root growth promotion by 20–40% (Xiang et al. 2017), the biochar effect on soil available P pool by 65% on average was definitely great. Yet, no negative change in soil available P was found as a single case in this study, compared to either positive or negative changes existed in all the soil attributes addressed in above-mentioned literatures. Thus, BSA had a promising but great potential to improve soil P availability in agricultural soils.

Soil P availability had been critical for use efficiency of P fertilizers in agriculture(Zhang 2016). In most soils, available P content was very low in the soil solution, represented less than 1% of total P and over 80% of soil P became immobile and unavailable for plant uptake because of adsorption, fixation, conversion of P to organic form, and precipitation (Norman et al. 1995; Vuuren et al. 2010). Soil available P pool following P fertilization depended mainly on reservation of available form in the soil and the capacity of soil absorbing P. Often, the soil capacity to reserve available form from the applied phosphorus fertilizers was low so that P supplied to soil became unavailable to plants (Hinsinger 2001). This study highlighted a profound potential of BSA to enhance P availability in agricultural soils. The great effect of BSA on soil available P obtained here was basically similar to the average effect of 68% for wide range of ecosystems by Biederman and Harpole (2013) but higher than the averaged effect size of 45% by Gao et al. (2019) in soils with 70 data pairs obtained from publications by 2017. However, Glaser and Lehr (2019) reported a positive but smaller increase in soil P availability in agricultural soils by ca 45% (reported as accumulation factor of 4.6) in a meta-analysis with 108 data pairs from studies published by 2016. The greater enhancement on soil P availability found with 516 data pairwise comparisons in present study further convinced a biochar’s role in enhancing soil supply of available phosphorus to plants and potentially improving P fertilizer efficiency in agriculture.

**Fig. 5:**
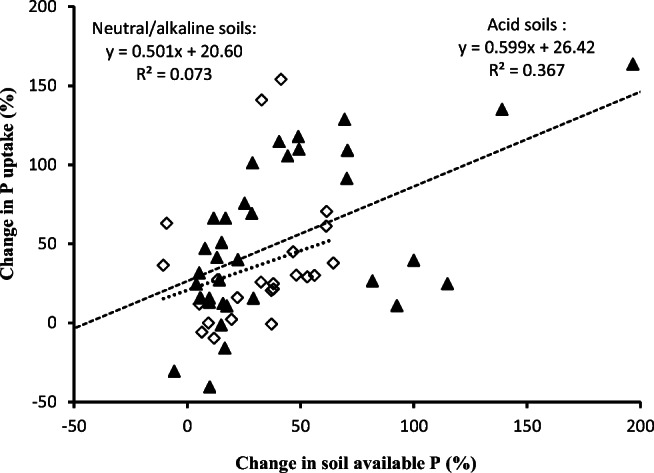
Change in plant P uptake related to changes in soil P level under biochar amendment in acid (▲, pH ≤ 6.5) and neutral/alkaline soils (◇, pH >6.5). The long dashed line and the short dashed line represents a significant correlation respectively for acid soils with pH ≤ 6.5 and for neutral/alkaline soils with pH >6.5

**Fig. 6:**
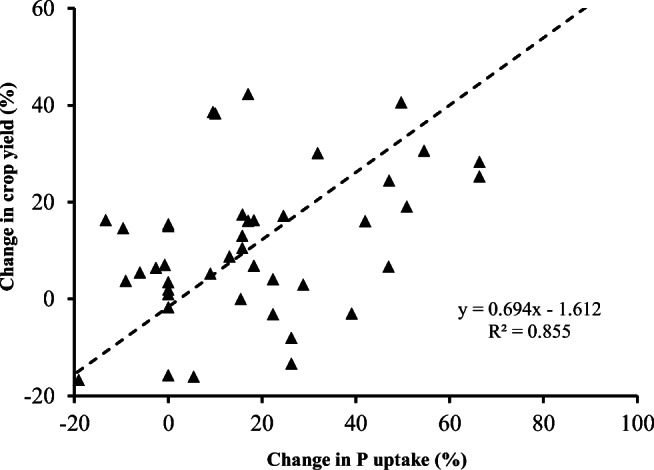
Change in crop yield as a function of changes in P uptake under biochar amendment

In our meta-analysis, biochar application showed an overall significantly positive increase in plant P uptake by 55% on average, being smaller than in soil available P (65% on average). With a moderate but significant correlation between the changes in soil available P and plant P uptake (Fig. 5), there were yet negative responses of plant P uptake to increase in soil available P. Furthermore, crop yield change with BSA, extracted from the reported studies, could be attributed by 54% to the positive change in plant P uptake (Fig. 6). This suggested a significant but weaker contribution by BSA to crop productivity improvement, compared to changes in soil available P and plant P uptake. Indeed, the BSA effect was well explicated as by 12.5% in plant biomass and by 11% in crop yield, in the work by Liu et al. (2013). Nevertheless, BSA-induced positive change in soil available P and plant P uptake could be a significant but moderate contributor for plant growth and productivity improvement, which could be affected by a variety of factors in agricultural soils (Jeffery et al. 2011). Generally, biochar application caused enhancement of P adsorption and utilization by plant, through improving soil conditions for promoting plant P uptake and assimilation such as plant root growth promotion (by 20–50%, Xiang et al. 2017) and soil microbial abundance (by 25%, Biederman and Harpole 2013). The latter would potentially contribute to utilization of soil inorganic or organic P through root exudates and microbial phosphorus enzyme activities (George et al. 2008; Giles et al. 2018). In their work for various ecosystems, Biederman and Harpole (2013) reported an average increase in plant P concentration by 20% despite of a net null in plant N. Yet, a general positive effect by biochar on plant N assimilation had been not yet clear though biochar increased N agronomic efficiency by ca 10% in Chinese rice paddies (Huang et al. 2013). In this study, the consistent but large increase in plant P uptake compared to biomass or yield enhancement pointed to a potentially increased recycle of P added to soil, of which only 8% was generally recovered in plant (Blackwell et al. 2015). Therefore, BSA appeared a helpful tool for increasing P use efficiency and recycling P in agricultural systems (Glaser and Lehr 2019) besides a potential P source as those from P-rich biomass feedstock (Zheng et al. 2013; Dai et al. 2016). Clearly, biochar role in P supply and crop production should be revisited beyond carbon sequestration and greenhouse gas mitigation (Kammann et al. 2017).

### Biochar effect on soil available P and plant P uptake: biochar recommendation

Either physical properties or chemical properties or both of biochars affected the effect size of BSA on soil carbon sequestration (Wang et al. 2017), greenhouse gas emission (Cayuela et al. 2013), soil nutrients (Biederman and Harpole 2013), metal mobility and stabilization (Chen et al. 2018), and soil hydrology (Omondi et al. 2016) as well as on microbial growth (Zhou et al. 2017). For crop productivity (Jeffery et al. 2011; Liu et al. 2013), however, plant type could be also a strong factor controlling biochar’s effect on plant growth and yield build-up. Yet, the biochar-induced changes in soil available P and plant P uptake could be driven by a number of key factors involved in soil physicochemical reaction and plant growth, in addition to improvement of soil fertility associated with SOM enhancement (Sohi et al. 2010; Amin and Eissa 2017) and soil reaction (Dai et al. 2017).

Properties of biochar had been often given priority in addressing biochar’s role in soil processes, particularly those of C content and stability (Wang et al. 2017; Chen et al. 2018), C/N ratio (Cayuela et al. 2013), and pH (Dai et al. 2017) as well as application dose (Ding et al. 2016). On soil available P pool in this study, biochar feedstock did not affect significantly the effect size but manure-derived biochar had higher effect size than wood biochar, probably due to the difference in P content and pH among the biochar types (Gaskin et al. 2008; Mullen et al. 2010; Uzoma et al. 2011). However, pyrolysis temperature mattered with the biochar effect on soil available P in a decreasing trend with increasing pyrolysis temperature( Cha et al., 2016; Uchimiya and Hiradate 2014). High temperature caused co-precipitation of phosphorus with inorganic minerals during pyrolysis (Novak et al. 2014; Ding et al. 2016; Gao et al.2019), leading to decrease in solubility of P in the produced biochars despite increased C stability (Lehmann and Joseph 2009). Thus, there could be a tradeoff between P solubility and C stability in biochar production in terms of pyrolysis temperature, when addressing biochar’s role in soil carbon sequestration (Wang et al. 2017).

**Fig. 7:**
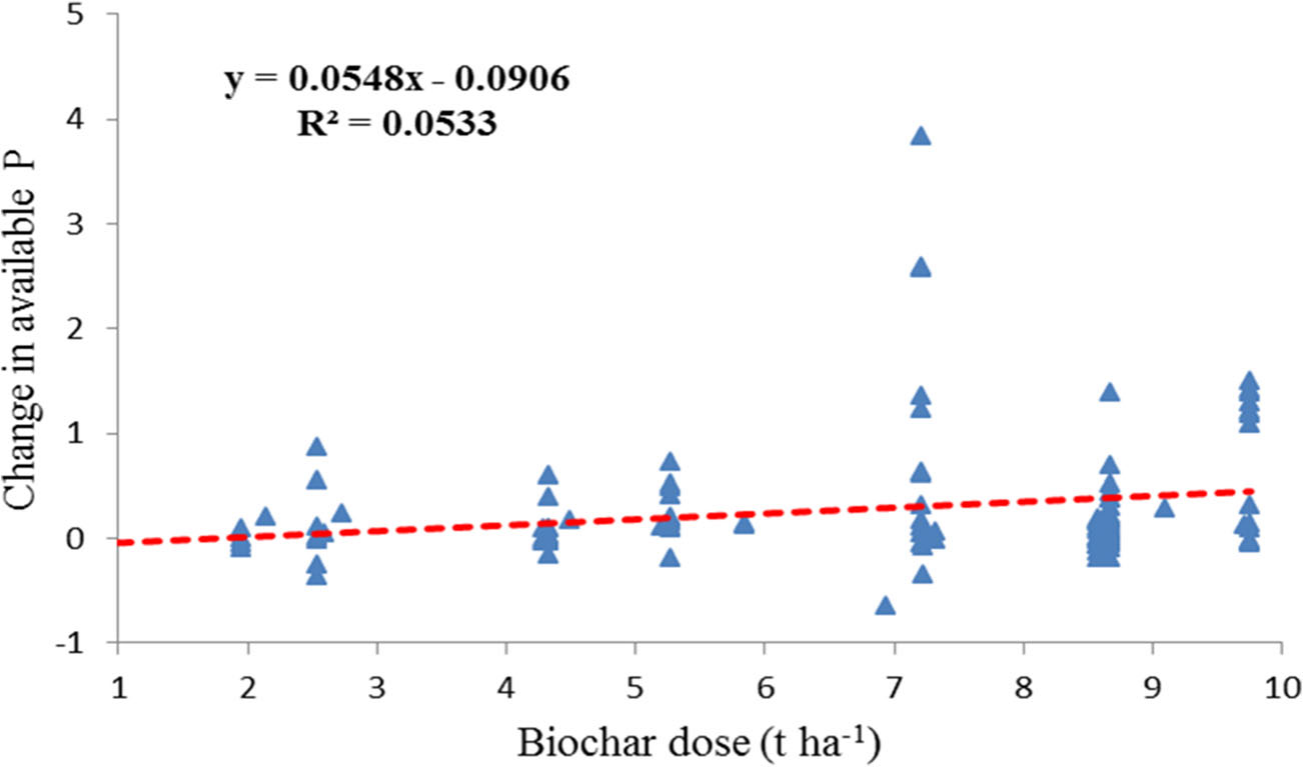
Change in plant P availability in amended soils as a function of biochar application rate when amended within 10 t ha^−1^ (R^2^=0.05 *p*<0.001)

Furthermore, the changes in soil available P with application rates were found similar to those in crop productivity, where excess application over 40 t ha^−1^ did not lead to increase in crop yield (Liu et al. 2013). This had been critically addressed as a cost-effective issue of biochar use in agriculture (Abbie et al. 2014, 2015) though soil available P increased with increasing biochar application rate up to 40 t ha^−1^ (Revell et al. 2012; Macdonald et al. 2014; Zhai et al. 2015; Jing et al. 2017). And the change in soil P availability was positively but very weakly correlated to biochar dose (Fig. 7) (*R*^*2*^ =0.05, *p*<0.05). Similarly, in the work by Glaser and Lehr (2019), plant-available P in biochar-treated soil was significantly but very weakly related to application rate. There were either consistent (Vanek and Lehmann 2015; Pandit et al. 2018 ) or inconsistent changes (Parvage et al. 2013; Kelly et al. 2015; Madiba OF et al. 2016; Abujabhah et al. 2016; Kizito et al. 2019) between soil available P level and biochar application rates. The plateau phenomena of biochar dose in terms of soil available P could be attributable to potential P immobilization via strong sorption onto the biochar surface (Kizito et al. 2019) or in co-precipitation in form of PO_4_^3−^ at high pH (Hass et al. 2012).

Unlike soil available P, changes in plant P uptake were generally regardless of biochar feedstock types, pyrolysis temperature, and the application dose (Fig. 2). Exemption was a significant increase with manure biochar and high dose over 40 t ha^−1^, probably due to the direct input in large amounts of soluble P from added biochar. For example, manure biochar contained as high as 19.9 g kg^−1^ of P and induced a 71% change in plant P of lettuce in 2% amended soil (Gunes et al. 2014). Increased plant P uptake could be attributed in part by biochar addition (by 20%, Shen et al. 2015) but largely due to soil-plant processes associated with P assimilation and yield build-up. As such, small yield change induced by biochar addition could not be relevant to a large change (by 65% in soil P availability, in this study). The failure of plant growth and yield at high dose (Uzoma et al. 2011; Borchard et al. 2014; Kloss et al. 2014; Laghari et al. 2015) could be explained with C/N ratio of biochar which could potentially cause N-deficiency to plants (Kloss et al. 2014; Uzoma et al. 2011). Nevertheless, application at higher rate would raise an economic barrier for farmer’s adoption to use biochar as a soil ameliorant in agriculture (Blackwell et al. 2010; Abbie et al. 2014, 2015). Instead, taking the advantage of improving soil P supply and plant uptake, biochar had been recently recommended to use for blending chemical nutrients as biochar compound fertilizer to shift chemical fertilizer paradigm (Joseph et al. 2014; Zheng et al. 2017). Overall, to use for soil fertility beyond SOM enhancement, low temperature biochar from manure at doses up to 20 t ha^−1^ could be chosen to use in agricultural soils.

### Biochar effect on soil available P and plant P uptake: soil recommendation

**Fig. 8:**
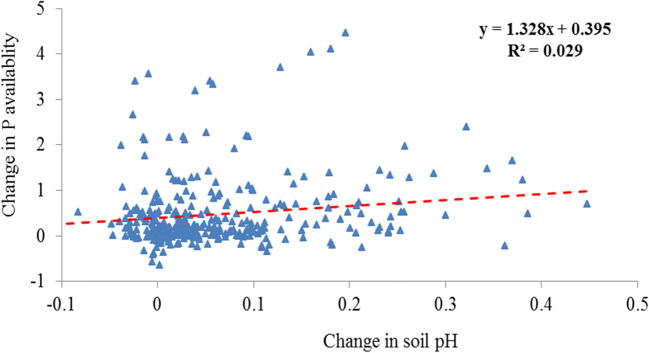
Change in soil P availability as a weak function of pH change in soil pH under biochar amendment (*R*^*2*^=0.03, *p*<0.001)

In soil amendment, biochar effects were basically via soil-biochar-plant interfaces process with soil properties as a determinant factor (Lehmann et al. 2015). In addition to biochar characters, soil factor could be a significant player in using biochar for improving soil P supply and crop productivity. In this meta-analysis, there were large variations of biochar’s effect on soil available P and plant P uptake in terms of soil conditions but of plant factor due to limited cases of plant categories. Changes both in soil available P and plant P uptake were found more or less negatively correlated to soil available P level and soil pH (Fig. 3). Both soil available P and plant P uptake was increased by almost one fold in P poor soils while by ca 40% in high P soils. Comparatively, increase in soil P and plant P uptake was by 80% in very acid soils (pH< 5.0) compared to by ca 40% in neutral soils. Moreover, a very significant correlation between soil P and plant P uptake was found in acid soils (pH≤ 6.5, *R*^*2*^=0.36, *p*<0.001) (Fig. 5) despite a significant but weak correlation between soil pH and soil available P (Fig. 8). In acidic soils, soil fertility could be mostly poor for phosphorus bound to hydroxides and oxides but unavailable to plant uptake (Ch'ng et al. 2014; Zhang et al. 2016b). In acid soils, moreover, biochar increased soil pH and buffering capacity (Chan et al. 2008; Uzoma et al. 2011), nutrient contents (Fu et al. 2012) and improved P mobility (Novak et al. 2009; Nigussie et al. 2012; Biederman and Harpole 2013) and microbial transformation (Warnock et al. 2007), and in turn P release to plant uptake (Shen et al. 2016). The finding here support the recommendation for priority use of biochar for improving soil fertility and crop production as well as SOC sequestration in acid poor soils (Lehmann 2006; Sohi et al. 2010; Zhang et al. 2016a, b).

Meanwhile, soil texture affected BSA’s effects both on soil available P and plant P uptake, which was higher in fine-textured soils than in coarse textured soils. This was similar to the report of a 25% higher effect on P availability in heavy textured than in sandy textured tropical Australian soils (Zhang et al. 2016a, b). Clearly, this seemed controversial to the finding by Liu et al. (2013), who noted a greater increase in crop productivity in sandy soils than in heavy soils. In the searched literature, heavy textured soils were often acidic and poor in P level (Figs. 5 and 8) with positively charged iron oxides minerals in their clay fraction. In such soils, amended biochar helped to elevate soil reaction and thus alleviate P immobilization so as to increase available P pool as well as augment of total P (Parvage et al. 2013; Novak et al. 2014; Wang et al. 2015; Dai et al. 2016). The more or less consistent change in soil available P and plant P uptake with BSA suggested that biochar use in acid heavy textured soils would be beneficial for improving soil P (Fig. 8) supply for potentially improving crop productivity, mostly in tropic regions with acid but P-poor soils.

## Conclusions

Overall, this meta-analysis allowed an extended understanding of changes in soil P availability and plant P uptake with biochar amendment to agricultural soils. With enhanced data of experiments, we could confirm a great biochar’s effect on increasing soil available P and plant P uptake by over 50%, being higher for any other effects by biochar in soil process and crop production reported so far. This study highlighted again an advantage of using biochar to improve soil supply of phosphorus, an issue of increasingly limited mineral resource beyond the issue of radiative N in world agriculture. Unlike carbon sequestration, biochar from manure and produced at low pyrolysis temperature significantly contribute to improvement of soil P availability and plant uptake. However, biochar exerted much greater improvement of soil available P and plant uptake in P-poor acid soils and heavy textured soils, being different from the effect for crop productivity. Clearly, there could be tradeoffs in biochar effects between carbon sequestration/gas emission, soil nutrient of N and P, and soil fertility/crop productivity. Further studies in field conditions should be deserved to provide insights into management of potential synergies/tradeoffs between productivity and climate change mitigation, between soil health and plant production.

## Supplementary Information

**Supporting material available online**: recorded data of retrieved experiments on soil and plant P under biochar soil amendment treatments.

Table S1 (DOCX 16 kb)

ESM 1 (XLSX 93 kb)
