# Supplementary material for: Could biochar amendment be a tool to improve soil availability and plant uptake of phosphorus? A meta-analysis of published experiments
Source: Environ Sci Pollut Res Int. 2021 May 8;28(26):34108–20. doi: 10.1007/s11356-021-14119-7 (PMC8275515; doi:10.1007/s11356-021-14119-7)
Supplement: Supplementary file 1 — (DOCX 16 kb) [file 11356_2021_14119_MOESM1_ESM.docx]

|  | Category | Soil available P only | Plant uptake only | Both soil P and plant uptake | Total/sum |
| --- | --- | --- | --- | --- | --- |
| Publication  year | By 2009 | 2 | 1 | 2 | 5 |
|  | 2010-2015 | 26 | 7 | 6 | 39 |
|  | 2016-2018 | 20 | 11 | 11 | 42 |
| Distribution | Asia/Pacific | 26 | 6 | 10 | 42 |
|  | Australia | 5 | 2 | 6 | 13 |
|  | Northern America | 9 | 3 | 1 | 13 |
|  | South America | 1 | 1 | 2 | 4 |
|  | Europa | 9 | 1 | - | 10 |
|  | Africa | 3 | 2 | - | 5 |
| Experiment type | Lab incubation | 21 | - | - | 21 |
|  | Pot | 14 | 7 | 16 | 37 |
|  | Field | 12 | 10 | 11 | 33 |
| Experiment length | <3 months | 20 | 7 | 10 | 37 |
|  | 3-9 months | 22 | 6 | 5 | 33 |
|  | 9-12 months | 7 | 2 | 3 | 12 |
|  | >12 months | 9 | 6 | 4 | 19 |

**Table S1** Information of the literature-extracted experiments used in the meta-analysis
